# Bioprinting vascularized skin analogs: a stepwise approach

**DOI:** 10.1093/burnst/tkaf018

**Published:** 2025-03-02

**Authors:** Linyang Liu, Eugenia Spessot, Khoon S Lim, Ziyu Wang, Suzanne M Mithieux, Devid Maniglio, Antonella Motta, Anthony S Weiss

**Affiliations:** School of Life and Environmental Sciences, University of Sydney, Building D17 New South Wales, 2006, Australia; Charles Perkins Centre, University of Sydney, Building D17, New South Wales, 2006, Australia; BIOtech Research Centre, Department of Industrial Engineering, Via delle Regole 101, 38123, University of Trento, Trento, Italy; Charles Perkins Centre, University of Sydney, Building D17, New South Wales, 2006, Australia; School of Medical Sciences, University of Sydney, Building D17, New South Wales, 2006, Australia; School of Life and Environmental Sciences, University of Sydney, Building D17 New South Wales, 2006, Australia; Charles Perkins Centre, University of Sydney, Building D17, New South Wales, 2006, Australia; School of Life and Environmental Sciences, University of Sydney, Building D17 New South Wales, 2006, Australia; Charles Perkins Centre, University of Sydney, Building D17, New South Wales, 2006, Australia; BIOtech Research Centre, Department of Industrial Engineering, Via delle Regole 101, 38123, University of Trento, Trento, Italy; BIOtech Research Centre, Department of Industrial Engineering, Via delle Regole 101, 38123, University of Trento, Trento, Italy; School of Life and Environmental Sciences, University of Sydney, Building D17 New South Wales, 2006, Australia; Charles Perkins Centre, University of Sydney, Building D17, New South Wales, 2006, Australia

**Keywords:** Bioprinting, Bioinks, Skin, Vascularization

## Abstract

Bioprinting has emerged as a promising technology for fabricating vascularized skin substitutes. The availability of functional skin tissue constructs is critical for the surgical treatment of various wounds, including ulcers and burns. Integrating functional vascular networks within engineered skin constructs is indispensable for ensuring adequate nutrient perfusion and overall tissue viability. This review undertakes a comprehensive exploration of the application of 3D bioprinting for fabricating vascularized skin tissue constructs. It encompasses an examination of the printing modalities, ink formulations, and cell-sourcing strategies currently prevalent in the field. The design and formulation of suitable inks are crucial steps in the successful bioprinting of vascularized skin constructs, and various ink components such as biomaterials, cells, growth factors, and bioactive molecules are particularly considered, with a focus on their roles in promoting angiogenesis and blood vessel formation within the printed constructs.

Highlights

- 3D bioprinting enables the fabrication of vascularized skin analogues through the precise layer-by-layer deposition of materials and cells.
- Design of ink formulations for vascularized skin analogues should consider material composition, cross-linking modifications, incorporation of angiogenic agents and heterogeneous cell sources to mimic the content of the natural extracellular matrix.
- Despite recent progress, challenges on the path to clinical translation include optimizing bioprinting techniques and ink formulations, achieving stable and functional vascular networks and scaling up bioprinted constructs.

## Background

Wounds caused by injury or disease can lead to skin tissue loss and extended healing even after treatment. Skin wounds can be classified as acute or chronic based on the cause and time taken to regenerate [1]. Autografts are the gold standard for treating severely injured patients for wound closure in a clinical setting [2]. However, the availability of autografts is limited, especially for treating large, injured areas. To address this issue, the field of skin tissue engineering has emerged and developed over the past few decades, demonstrating great potential as an alternative treatment to improve wound healing and tissue regeneration. Commercialized avascular skin substitutes, such as Integra, Dermagraft, and Apligraf, have been widely investigated for treating acute and chronic wounds [3]. They rely on endogenous vascularization and subsequent remodeling after implantation to impart viability. However, the initial vascularization phase can be prolonged, impacting the overall healing outcome [4, 5]. In addition, human skin has a complex structure with three major layers and multiple cell types [6]. Therefore, for accelerated repair, it is crucial to consider vascularization in a functional 3D tissue analog [7].

Developing biomimetic vascularized tissue is challenging. Tissue engineers have sought to vascularize tissue analogs *in vitro* by recapitulating *in vivo* vascularization processes [8]. *In vivo*, vascular network formation and remodeling are multifaceted processes that require the coordinated recruitment of multiple cell types regulated through biochemical (e.g. angiogenic factors), biophysical, and mechanical cues from the surrounding microenvironment [9–11]. Blood vessel network formation *in vivo* occurs primarily through vasculogenesis and angiogenesis. Vasculogenesis is the process of *de novo* blood vessel formation, through the self-assembly of endothelial cells (ECs), including those derived from endothelial progenitor cells, into vascular networks [12]. Angiogenesis refers to the formation of new vessels from pre-existing blood vessels through the process of sprouting [13]. Angiogenesis is stimulated through a demand for oxygen and nutrient delivery to tissue undergoing hypoxia [14, 15]. In hypoxia or upon injury, the release of bioactive molecules in the vascular endothelial growth factor family [16] and mechanical cues activate quiescent ECs to remodel the vasculature and expand the vascular bed [16, 17]. Activated ECs proliferate and differentiate into tip and stalk cells with specialized phenotypes; the tip cells perform invasive and migratory functions, mainly through the production of matrix metalloproteinases that degrade the basement membrane surrounding the vessel [18] to allow branching from the pre-existing vasculature. Guided by these tip cells, stalk cells elongate and proliferate to create lumenized sprouts [10] that are stabilized by cell–cell communication between ECs and recruited parenchymal cells. The sprouts then anastomose to neighboring vessels to form new vascular networks [19]. Tissue perfusion by blood flow further stabilizes and gradually expands the newly formed vascular network.

Vascularization of tissue-engineered skin is generally achieved through bottom-up or top-down fabrication [20]. Bottom-up fabrication mimics the *in vivo* vasculogenesis and angiogenesis processes, relying on angiogenic factors to induce seeded ECs to form new vasculature in scaffolds or hydrogels [21]. The vasculature is then matured *in vitro* (for example, in bioreactors) to form stable and functional networks [21, 22]. Top-down fabrication relies on pre-vascularization *in vitro*, typically achieved by creating pre-designed, interconnected, and hollow microchannels within the tissue construct, followed by cell seeding into the microchannels. Sacrificial materials are commonly used to create the hollow structures [23]. Typically, however, both these approaches neglect the importance of establishing an extracellular matrix (ECM) environment that mimics skin tissue to enable cellular functions that are critical for stable vascular network formation. This requires the skin constructs to have suitable architectural elements, including morphology and porosity, biologically relevant ECM-mimicking material complexity, and physiological swelling behavior. They should also include spatially arranged biochemical and physical cues to guide EC adhesion, proliferation, migration, and connection [24, 25].

3D bioprinting is the ideal approach to satisfy these requirements as it allows for precision architectural control, multi-material printing, and spatial arrangement of cells and bioactive molecules. It has great potential for creating the complicated three-layered and vascularized architectures required for a mimetic functional skin tissue [26]. Organized vascular networks can be fabricated by strategically creating connected channels in printed constructs to ensure adequate perfusion and avoid hypoxic regions. 3D bioprinting can also solve the limitations in traditional *in vitro* skin models by offering customized construct sizes and shapes, lower processing costs, and higher reproducibility [6, 27].

**Figure 1:**
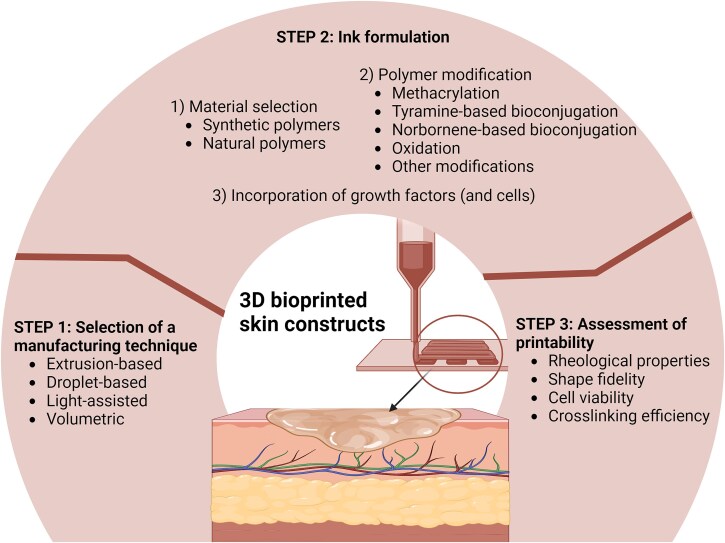
Design of inks for 3D bioprinted skin constructs—a multistep approach. Figure was created with BioRender.com

3D bioprinting requires a stepwise approach that includes the selection of printing technique, ink formulation, and assessment of printability (Figure 1) [28]. Among them, ink design plays a pivotal role in generating vascularized skin tissue analogs, as it directly influences the structural properties of the bioprinted constructs and their ability to support cell growth and function effectively [29]. Printable inks that contain biomaterials mixed with cross-linking agents are referred to as biomaterial inks, while cell-containing inks are referred to as bioinks [30]. These two types of inks exhibit distinct physical, chemical and mechanical properties.

This review discusses the stepwise process of bioprinting vascularized skin tissue analogs, with a focus on designing optimal bioinks. It also presents an overview of advances and challenges currently faced in the field.

## Review

### Bioprinting techniques

This section briefly introduces a variety of 3D bioprinting techniques that can be considered for the construction of vascularized skin analogs.

#### Extrusion-based bioprinting

Extrusion-based bioprinting typically uses pneumatic pressure to deliver inks through one or multiple nozzle printheads onto a platform or within a suspension bath [8, 31]. As it uses a relatively simple air pressure-driven printing technique, it can also be performed with a handheld device to achieve *in situ* printing directly onto a wound area [32]. When the viscosity of the ink is low, it can be extruded within a supporting bath, in a process called free form bioprinting [33–36]. The supporting bath is viscous to prevent the collapse of mechanically weak extruded bioinks, allowing complex structures to be formed. The use of a supporting bath also limits dehydration, which is an inherent disadvantage of printing directly onto a platform [31]. After printing, the supporting bath can be washed away, leaving the printed construct for further processing. Gelatin [33, 34] and cross-linked alginate [37] have been investigated as supporting bath media. Although freeform printing has been used to create millimeter-wide hollow tubular structures within porous cell-laden hydrogel constructs [38], capillary-like vascular networks require higher channel resolution down to the micrometer scale making tube diameter a potential limitation of this form of printing.

#### Microfluidics bioprinting

Microfluidic bioprinting uses a microfluidic device as the printhead, in which one or multiple inks flow through microchannels that precisely control the flow rate, pressure, and moving direction [39, 40] of the inks. This enables the strategic manipulation of inks, allowing for them to either stay in layers or mix with other inks or cells [41–44], followed by deposition with high spatial and temporal accuracy. Therefore, microfluidics bioprinting can be used for printing heterogeneous skin tissue analogs with simultaneous multi-ink and cell deposition allowing for bio-functionality such as vascularization [45]. Furthermore, the ability to extrude inks at low viscosity or in a liquid form using a coaxial flow-focusing device assists in maintaining cell viability. Coaxial flow-focusing printheads can also be used to print sacrificial inks such as pure gelatin and wax, which can be washed away after printing, leaving hollow channels to mimic vascular networks for further cell seeding [43, 46, 47].

#### Droplet-based bioprinting

Droplet-based or drop-on-demand bioprinting was developed from inkjet printing, where inks are deposited in droplet form onto a designated supporting substrate. The droplets can be formed in different ways, such as through the use of thermal or piezoelectric actuators, electrostatic forces, acoustic waves, or solenoid magnetic fields [48]. Droplet-based bioprinting is affordable, precise, and has great potential for *in situ* clinical printing applications [49]. However, the applied voltage and other related process parameters, such as nozzle orifice size, temperature, and selected actuation mechanism, can impact post-bioprinting cell viability. The printing nozzle is prone to clogging, limiting material selection and cell densities to fewer than 10 million cells/mL [50]. Furthermore, the ejected droplets may gel quickly in the air, preventing them from assembling into the desired 3D structure. Droplet-based bioprinting is also not ideal for printing porous structures, limiting its application in skin tissue engineering.

#### Light-assisted bioprinting

Light-assisted bioprinting includes laser-assisted bioprinting (LAB), stereolithography (SLA), and digital light processing (DLP) bioprinting [51–53]. LAB uses laser beams to precisely melt cell-containing sheets or hydrogels and deposit the material onto an underlying substrate with high resolution [54]. LAB can be used to print multicellular components and create hollow channels. Although LAB is free from nozzle clogging, it has been limited by potential photonic cytotoxicity and scalability issues in vascularization applications [54]. SLA and DLP bioprinting have recently attracted more attention in producing layered structures. They use a projected laser or digital light to solidify bioinks layer-by-layer, to form cured 3D constructs from a photo cross-linkable precursor solution [55, 56]. SLA/DLP bioprinting provides a fast and cell-compatible way to spatially fabricate tissue constructs in high resolution (down to 10 μm) [53, 56, 57]. Although light-assisted bioprinting is still in its developmental phase, it holds great potential for fabricating cell-dense and structurally complex constructs with high resolution and mechanical and chemical gradients at the microscale. The major limitation of light-assisted bioprinting is that ink selection is restricted to photosensitive polymers, which in most cases, must be created through chemical modification.

#### Volumetric bioprinting

Volumetric bioprinting (VBP) is an emerging technique that uses tomographic projections to rapidly solidify photosensitive inks into complex, centimeter-scale tissue constructs [58–60]. In VBP, a physically cross-linked hydrogel containing a photo cross-linking reagent is connected to a rotating platform. A light source with a spatial light modulator is used to deliver filtered tomographic back-projections to the hydrogel, and thus, a 3D anisotropic structure is generated with 3D light-dose distribution [60]. It avoids the limitation of traditional 3D printing that relies on slicing software that generates G-code to instruct the printhead movements layer-by-layer [28]. VBP is an ultra-fast process that can take from seconds to tens of seconds compared with traditional extrusion-based bioprinting (minutes), with equal or higher resolution down to 40 μm [58, 60]. Selection of the photo cross-linkable hydrogel material is critical in VBP. Key physicochemical features such as viscosity, transparency, and photo cross-linking kinetics will determine the printing time, resolution, cell viability, and photo initiator concentration required [61]. VBP is a promising technique for fabricating tissue mimics in clinically relevant sizes with high cell viability [62].

### Ink formulation

Selecting appropriate inks for bioprinting vascularized skin analogs is a complex process that significantly influences the printability and biological performance of the final constructs. In general, the ink needs to contain cross-linkable hydrogel precursors and biochemical cues that can be formed into 3D structures with sufficient mechanical strength to maintain structural integrity, a suitable degradation profile, and an ECM-like configuration that can support skin cell proliferation and migration, blood vessel formation and tissue regeneration [63–65]. This section discusses polymers and growth factors that have been selected as ink components for the construction of skin analogs and the cross-linking mechanisms that have been utilized.

#### Ink selection

To bioprint vascularized skin analogs, inks that are composed of natural polymers such as alginate, hyaluronic acid (HA), gellan gum, collagen, gelatin, tropoelastin, and silk are preferred over synthetic polymers as they retain bioactive motifs and signaling cues that can mimic the native ECM [66]. However, natural polymers are limited by insufficient functional groups for cross-linking and poor mechanical properties. To solve this issue, natural materials can be chemically functionalized or mixed with other materials to achieve desirable mechanical properties, degradation rates, and structural characteristics, making them suitable for elastic and durable skin analogs.

##### Alginate

Alginate, one of the most common natural polymers used in bioprinting, is an anionic polysaccharide extracted from brown algae. It consists of β-D-mannuronic acid (M block) and α-L-glucuronic acid (G block) units [67, 68]. Alginate can be easily dissolved in aqueous media and ionically cross-linked to form hydrogels through interactions with divalent cations such as Ca^2+^, Sr^2+^, and Ba^2+^ [69]. Alginate-based inks can efficiently encapsulate skin cells and are compatible with different bioprinting techniques, such as inkjet [70, 71] and extrusion-based techniques [72, 73]. To achieve better bioactivity, alginate is commonly cross-linked with other natural materials, such as gelatin. A double cross-linked 3D hydrogel containing hydroxyphenyl propionic acid (HPA) conjugated gelatin and tyramine-modified alginate that was extrusion bioprinted with human umbilical cord mesenchymal stem cells (MSCs) showed faster wound healing rate (after 3 days of treatment) with an improved wound healing effect *in vivo* [73]. Crosslinked alginate can also improve the mechanical strength of bioprinted constructs [74]. The molecular weight of alginate and the ratio between M and G blocks (M/G ratio) impact an alginate-based hydrogel mechanical properties and its degradability [75]. By varying these factors, hydrogels with tunable stiffness can be obtained, which is beneficial for culturing ECs toward vascularization [76].

##### Hyaluronic acid

HA, a natural polysaccharide found in skin ECM, is widely used as a bioink component due to its ability to promote wound healing and skin tissue morphogenesis [77–85]. It is composed of D-glucuronic acid and N-acetyl-D-glucosamine subunits and HA-based inks can be printed and cross-linked through physical or chemical mechanisms [86, 87]. HA can also be modified and cross-linked with other biomaterials to obtain improved physiochemical properties. For example, N-(2-aminoethyl)-4-(4-(hydroxymethyl)-2-methoxy-5-nitrosophenoxy) butanamide (NB) linked HA and gelatin methacrylate (GelMA) was mixed with human skin fibroblasts and human umbilical vein endothelial cells (HUVECs) for DLP bioprinting [57]. The bioink was crosslinked under UV light with lithium phenyl-2,4,6-trimethylbenzoylphosphinate (LAP) to form an adhesive layered structure. The *in vivo* dermal regeneration and neovascularization potential were analyzed by immunostaining for EC markers, encompassing cluster of differentiation 31 (CD31) and vascular endothelial growth factor receptor 2 (VEGFR-2) on day 21.

##### Gellan gum

Gellan gum is an anionic polysaccharide composed of repeating units of a tetrasaccharide comprising α-L-rhamnose, β-D-glucuronate, and two residues of β-D-glucose. It exhibits thermoresponsive characteristics, efficiently forming a gel at physiological temperatures. Due to these favorable rheological properties, it has been used as a bioink material and shown cytocompatibility following extruding and culturing of human keratinocytes (HaCaT) for 7 days [88]. However, since gellan gum lacks cell-binding motifs and has relatively low mechanical strength, it is often mixed with other ink materials, such as gelatin, when used for functional skin applications [89].

##### Collagen

Collagen, a major component of skin tissue with a triple helix structure, is ideal for biomaterial inks due to its mechanical strength and arginyl-glycyl-aspartic acid (RGD) motifs that promote cell attachment and proliferation [90–93]. However, collagen molecules self-assemble into fibrils and form a gel under physiological conditions, making pure collagen challenging to extrude. Therefore, it is usually modified [94–96] or blended with other polymers, such as HA [97] or GelMA [98], to form biomaterial inks, obtaining enhanced viscoelasticity for printing and proper degradation rate. For example, recombinant human type III collagen (rhCol3) was mixed with GelMA and human dermal fibroblasts (hDFs) to form a composite bioink for extrusion-based bioprinting. HaCaTs were then seeded on the printed construct to create a 3D skin tissue analog *in vitro*. rhCol3 within the bioink improved HaCaT proliferation and migration at an early stage *in vitro* (3 days) and further enhanced the wound healing process after 14 days *in vivo* [98].

##### Gelatin

Gelatin is derived from collagen through partial hydrolysis but retains the RGD cell-adhesive sequence. This makes it attractive to develop bioinks that support the activities of various wound healing-related cells, including ECs, fibroblasts, and stem cells [99, 100]. Gelatin is often chemically modified with methacrylate groups to form GelMA to allow photo cross-linking, ensuring the stability of printed skin constructs [101]. GelMA has been blended with modified ECM components, such as methacrylated tropoelastin (MeTro). Tropoelastin is an essential component of soft tissues such as skin, where it imparts elasticity and has fundamental roles in cell migration and angiogenesis [102, 103]. Lee *et al*. investigated a GelMA/MeTro bioink to fabricate a vascularized soft tissue using extrusion-based bioprinting, which delivered endothelium barrier function and spontaneous beating of cardiac muscle cells. The printed construct was also attractive because it displayed a minimal inflammatory response and was efficiently biodegraded *in vivo* subcutaneously in rats [104]. Gelatin can also be used as a sacrificial material due to a sol–gel transition at 28–34°C, allowing physical gelation below room temperature [27, 105]. Thus, it can be printed and processed at lower temperatures, and dissolved and washed away when the temperature is increased to physiological levels, leaving hollowed structures for nutrient exchange and vascularization [74].

##### Silk fibroin

Silk fibroin (SF), a protein extracted from *Bombyx mori* silkworm cocoons, can be processed into an aqueous solution for bioprinting applications [65, 106–109]. Its molecular weight and mechanical properties vary depending on the degumming method and dissolution protocol. This can broadly impact the quality and functionality of the printed construct. SF contains reactive functional groups, which enables various cross-linking methods to form stable hydrogels after printing. Physical inducers during or after bioprinting, such as introducing shear stress by extrusion of SF from printer needles, vortexing, changing temperature, and varying pH during printing, can speed up the β-sheet formation within the silk fibroin structures. This will allow the self-assembly of the SF to form stable and dense hydrogel structures. Apart from its use in extrusion-based bioprinting, SF-based inks have also been explored in DLP and VBP [106]. For example, an *in vitro* full-thickness skin wound model was 3D-printed by DLP using a methacrylated SF-gelatin ink, which provided a suitable environment for normal epidermal keratinocyte and fibroblast growth [52].

##### Fibrinogen

Fibrinogen is a soluble glycoprotein found in blood, which can be converted into a fibrous 3D matrix called fibrin mainly through enzymatic reactions catalyzed by thrombin [110, 111]. It is a beneficial component of biomaterial inks due to its ability to stimulate angiogenesis [112]. Fibrinogen was used as a bioink material with collagen, autologous dermal fibroblast and epidermal keratinocytes for *in situ* inkjet bioprinting. Wounds treated with the bioprinted autologous cell-containing hydrogel showed complete wound closure in 8 weeks, reduced wound contraction and increased re-epithelialization in porcine models [49]. However, fibrinogen alone is unsuitable for extrusion-based bioprinting due to its Newtonian behavior, and normally needs to be combined with other biomaterials such as gelatin, GelMA, or alginate to fabricate tissue analogs for vascularized skin regeneration applications [113–115].

##### Sacrificial inks for bioprinting vascularized skin analogs

Apart from ink components that will form the stabilized hydrogel construct, the fabrication of vascularized skin analogs using the top-down method requires sacrificial support materials that can be washed away after printing, leaving behind desired shapes or hollowed-out vascular-like networks within the construct.

Various biocompatible materials, such as alginate [116], agarose [117, 118] and some synthetic polymers, including polyvinyl alcohol (PVA) [119], polycaprolactone (PCL) [120, 121] and Pluronic F127 [122, 123] can be used as sacrificial ink components. These materials often possess high solubility in water or thermoplasticity and can therefore be easily removed through temperature changes.

Among them, PCL, a thermoplastic polymer that is used in a range of FDA-approved products, is often used as a robust supporting structure for bioprinting soft material [124]. For example, a full-thickness skin equivalent was fabricated by embedding a 3D-printed PCL mesh in a layer of acellular collagen and then sequentially depositing two different bioinks loaded with hDFs and HaCaTs onto the construct *via* extrusion bioprinting [120]. In another study, PCL was 3D-printed as a supportive mesh for dispensing collagen bioink loaded with hDFs. Inkjet bioprinting was then used to distribute epidermal keratinocytes uniformly onto the collagen matrix. The morphology of the engineered construct resulted in an *in vitro* skin model that resembles native skin in histological appearance [121].

#### Modification for cross-linking: side groups matter

To increase the number of polymers that can be considered as ink components, chemical modification is used to introduce functional groups to the polymer backbone chain, to allow various cross-linking methods such as UV light, chemicals, enzymes, or temperature to be used [125]. Customized chemical modification also allows for the control of cross-linking kinetics and the tailoring of mechanical properties for printed skin tissue analogs [126]. Common polymer modification reactions for fabricating soft tissues include methacrylation, tyrosine bioconjugation, norbornene bioconjugation, oxidation reactions, host-guest chemistry (i.e. β-cyclodextrin and adamantane), and multistep functionalizations. Table 1 summarizes examples of different modification reactions used for bioprinting soft tissues to promote vascularization and wound healing [127–131].

**Table 1:** Examples of polymer modification reactions for designing bioinks to promote vascularization and skin wound healing

**Polymer modification reaction**	**Polymers**	**Cells**	**Printing technique**	**Type of cross-linking**	**Structure + resolution**	**Biological outcome**
Methacrylation	Hyaluronic acid	Human umbilical cord MSCs	Extrusion	Photo cross-linking with Irgacure2959 (0.1% w/v)	Filaments with diameter 176 +/− 32 μm	Reduced inflammation, fast reconstruction of the ECM, and acceleration of the angiogenesis process were shown in mice [127].
	Silk fibroin/gelatin	HUVECs and hDFs	DLP	Photo cross-linking with LAP (0.3% w/v)	Porous construct pore sizes up to 100 μm	3D artificial skin model with multiple layers showed *in vitro* cell proliferation within the inks, particularly HUVECs and hDFs in the prevascularized layer, hDFs in the dermis layer, and normal human epidermal keratinocytes in the epidermis layer [52].
	GelMA/rhCol3	HDFs and HaCaTs	Extrusion	Photo cross-linking with LAP (0.25% w/w)	n.d.	HDFs within the constructs showed over 90% viability for 14 days with a spread morphology *in vitro*. The proliferation and migration of HaCaTs were improved in the presence of rhCol3. Wound healing was observed on the dorsal surface of rats [98].
	SilMA/GelMA	Mouse fibroblasts	Extrusion	Photo cross-linking with LAP (0.25% w/w)	n.d.	The bioprinted skin patch supported fibroblast proliferation for up to 7 days *in vitro* and promoted skin wound healing in mice [128].
	HAMA	HUVECs	DLP	Photo cross-linking with LAP (0.05% w/v)	Channels with diameters up to 360 μm	Within the bioprinted vascular analog, HUVECs exhibited an elongated morphology and expressed endothelial marker CD31 over 28 days of culture *in vitro* [129].
Tyrosine bioconjugation	Tyramine-modified alginate/hydroxyphenyl propionic acid-conjugated gelatin	Mouse fibroblasts and human umbilical cord MSCs	Extrusion	Photo cross-linking with Ru/SPS (0.5/10 mM)	Strands with diameters of ~500 μm	After implantation in mice, wounds treated with the bioprinted cell-encapsulated hydrogel construct formed an intact epidermal layer with vessel-like structures. The construct promoted angiogenesis and wound closure *in vivo* over 14 days post-implantation [73].
Norbornene bioconjugation	Gelatin	HUVECs and MSCs	DLP	Thiol-ene (thiol-norbornene) cross-linking with Ru/SPS (0.5 mM/5 mM)	Strands with diameters up to 200 μm	DLP bioprinting was used to create channels within the bioprinting hydrogel, and microcapillaries were assembled *in vitro* by coculturing HUVECs and MSCs for 7 days [130]. This demonstrated the potential of using norbornene-based photo cross-linking for vascularized skin analogs.
	Collagen	hDFs and HUVECs	Extrusion/SLA	Thiol-ene (thiol-norbornene) cross-linking with LAP (0.025% w/v)	Filaments with diameters of ~200 μm	Cross-linked norbornene-functionalized collagen demonstrated vascular network formation *in vitro*, providing a potential solution for vascularized skin analogs [96].
Oxidation reaction	Alginate	Mouse fibroblasts	Extrusion	Schiff-base reaction	Strands with diameters of 265 μm	The cross-linked hydrogel showed good viability (~80%) of fibroblasts for up to 21 days *in vitro* and has the potential for skin regeneration [131].

*HAMA* methacrylated hyaluronic acid, *n.d.* not determined, *SPS* sodium persulfate, *MSCs* mesenchymal stem cells, *HUVECs* human umbilical vein endothelial cells, *hDFs* human dermal fibroblasts, *SLA* stereolithography, *DLP* digital light processing, *ECM* extracellular matrix

##### Methacrylation

Methacrylation reactions modify polymer chains by introducing methacrylate groups onto amine (-NH2), carboxyl (-COOH), and hydroxyl (-OH) groups. The reaction can be carried out using either methacrylic anhydride or glycidyl methacrylate. Incorporation of the methacrylate groups allows for UV light-based crosslinking of the polymer. Many methacrylated polymers have been developed as components of biomaterial inks for skin/soft tissue bioprinting, including GelMA [128, 132], methacrylated hyaluronic acid (HAMA) [132, 133], methacrylated silk fibroin (SilMA) [134, 135], MeTro [104, 136], and methacrylated alginate [137]. For example, GelMA/HAMA bioinks were developed for creating stable, perfusable, EC-lined channels *in vitro* using SLA bioprinting [129].

##### Tyrosine bioconjugation

Tyrosine-based cross-linking relies on the oxidation of phenolic groups in tyrosine residues, resulting in the formation of dityrosine chemical bonds [138]. Natural tyrosine-rich polymers, such as HA [139, 140], alginate [141] and gelatin [142] can undergo this cross-linking reaction. Additionally, tyramine groups can be conjugated to polymer backbones through 1-ethyl-3-(3-dimethylaminopropyl)carbodiimide/N-hydroxysuccinimide (EDC/NHS) coupling reactions [143] or *via* DMTMM (4-(4,6-dimethoxy-1,3,5-triazin-2-yl)-4-methylmorpholinium chloride) amidation [144] to facilitate cross-link formation. The cross-linking can be initiated either by enzymatic catalysts such as peroxidase, tyrosinase, and laccase or through photo radical activation using photo initiators including ruthenium (Ru), riboflavin, and rose bengal [73, 97, 145, 146]. Tyramine-conjugated polymers can be used to design pre-cross-linking reactions (e.g. enzymatic) with controlled kinetics to improve the printability of certain materials [147] or to undergo gelation in a supporting bath containing cross-linking reagents after printing [129].

##### Norbornene bioconjugation

Norbornene, a cyclic alkene, offers a versatile method for cross-linking natural polymers through thiol-norbornene photo cross-linking reactions. This involves introducing norbornene groups into polymers *via* amide reactions, which can then form cross-links with thiol-containing cross-linkers under UV light exposure [148]. Thiol-norbornene photo cross-linking reaction can be initiated by different types of photo initiators including LAP, Irgacure 2959 [96], eosin-Y and Ru [73]. This reaction is highly efficient even at low concentrations of polymer, radical species, and degrees of substitution, making it suitable for bioink formulations and cell encapsulation. It overcomes the limitations of methacrylated polymers, which require high concentrations of propagating radicals that can damage encapsulated cells [61, 149, 150]. Norbornene-conjugated biomaterial inks such as gelatin-norbornene and polyethylene glycol (PEG)-norbornene have been widely adopted in various bioprinting techniques, including extrusion-based, DLP, and VBP [53, 61, 151]. It was demonstrated that a gelatin-norbornene bioink containing human stromal cells and ECs could be extruded and cross-linked into 3D constructs where ECs assembled into microcapillaries *in vitro* [130], while a PEG-norbornene biomaterial ink could be used to print, through DLP, perfusable microchannels that supported EC endothelialization [151].

##### Oxidation reactions

Oxidation is usually achieved by introducing aldehyde groups into polymer chains through the use of periodate. This process has led to the development of oxidized alginate [152] and oxidized HA [153]. The resulting aldehyde groups are highly reactive and can covalently bind with free amino groups present in other materials, such as gelatin [154], silk fibroin [131, 155] and chitosan [156], resulting in Schiff base formation.

##### Host-guest chemistry

In extrusion-based bioprinting, it is important to design inks with tunable rheological characteristics so that the ink material can be extruded smoothly through the nozzle while maintaining shape after printing. The host-guest chemistry involves a non-covalent reversible interaction between larger host molecules and smaller guest molecules, forming shear-thinning and self-healing materials [157, 158]. These properties have made it attractive in the bioprinting field. A typical example is the interaction between β-cyclodextrin (β-CD) and adamantane (Ad) [159], which can be chemically bound to natural polymers *via* amine bonding and esterification, respectively [160]. Studies have shown that by modifying HA, supramolecular interactions formed rapidly upon mixing CD-HA and Ad-HA through reversible host-guest bonds between β-CD and Ad moieties [158]. The reversible host-guest bonds can be disrupted by physical stimuli, such as shear stress, and rapidly recover upon removal of the shear stress. This behavior makes the material self-healing [161]. However, the host-guest bonds are not mechanically strong, which requires another type of covalent cross-linking to form the final bioprinted product [162, 163]. For example, in addition to using host-guest bonds between isocyanatoethyl acrylate modified β-cyclodextrin β-CD-AOI_2_ and acryloylated tetra-ethylene glycol-modified adamantane (A-TEG-Ad), a secondary covalently linked hydrogel network was formed by photo cross-linking between the modified host-guest supramolecule and GelMA. This biocompatible ink showed both shear-thinning and self-healing properties with an improved compression modulus from 0.1 to 0.6 MPa [163].

##### Multistep functionalization

Multistep functionalization of ink materials can achieve multi-cross-linking reactions during printing. It combines chemical modification, self-assembly, and photo cross-linking to improve the processability of certain polymers, particularly for enhancing printability in extrusion-based bioprinting. This strategy has been adopted for a thiolated Pluronic F127, methacrylated HA and human umbilical cord MSC containing bioink. Pre-cross-linking was carried out through a Michael addition reaction at a low temperature (4–20°C) followed by self-assembly at a higher temperature (30–37°C) and a final photo cross-linking triggered by a thiol-ene reaction. The bioink was able to promote full-thickness wound healing in mice from 7 days post-implantation [127].

#### Incorporation of angiogenic agent

3D bioprinting can provide spatial cues that guide cell behavior and tissue formation by precisely stacking and assembling tissue layers and vascular network-like microchannels [164]. However, the choice of bioink is confined to the specific requirement of the printing technology and often lacks bioactivity. To address this limitation, angiogenic factors such as VEGF [24, 25, 32, 165], platelet-derived growth factor (PDGF) [166], and basic fibroblast growth factor (bFGF) [167] are often added to the bioink to improve the biological outcome by inducing EC vasculogenesis and angiogenesis *in vivo* [16, 168, 169]. These angiogenic factors can be incorporated into the bioink by direct mixing with the pre-gel materials [170] or by immobilizing them onto the bioink polymeric chain through molecular bonding [171]. Pre-mixing with the bioink is the simplest way to stimulate EC migration, proliferation, and interaction [172]. However, chemical conjugation allows sustained release of angiogenic factors during matrix degradation. These methods can be used separately or together to achieve vascularization in bioprinted skin tissue analogs [173]. Apart from growth factors, one study demonstrated that copper oxide nanoparticles incorporated in a biomaterial ink could promote angiogenesis *in vitro* by regulating reactive oxygen species (ROS) generation [174].

#### Cell sources for vascularized skin analogs

Cell selection is another critical factor to consider when bioprinting functionalized skin equivalents. Skin, as the largest organ of the human body, has a complex, layered structure and high cell heterogeneity [175]. The skin epidermis comprises mainly keratinocytes and some melanocytes, while the highly vascularized dermis layer contains dermal fibroblasts, ECs, smooth muscle cells, and immune cells. The hypodermis contains adipocytes and stem cells [167].

In skin bioprinting, keratinocytes and fibroblasts are the two most used cell types [49, 57]. Keratinocytes produce keratin and regulate the skin barrier function [176, 177], while fibroblasts deposit ECM components such as collagen and elastin [178]. They are often printed and cocultured in different layers to form complex skin-like structures for use in wound healing applications [52, 179, 180]. To support both cell types in this type of system, the culture medium is often made from a 1:1 mixture of both fibroblast and keratinocyte media [181]. Additionally, keratinocytes are generally seeded on the top layer to ensure air-liquid conditions which promote their differentiation [52, 179, 180]. Although bioprinting keratinocytes and fibroblasts can produce layered epidermal- and dermal-like structures, the process is not sufficient for building the complex vascularized environment of the skin. ECs are pivotal in developing vascularized skin constructs. *In vivo*, angiogenesis occurs *via* communication and interactions between ECs and various types of parenchymal cells, which can also be referred to as perivascular cells. Parenchymal cells support the growth and migration of ECs while adding stability to the vessel network. To better mimic this *in vivo* scenario, coculturing ECs with different parenchymal cells using finely adjusted seeding densities [182] and ratios [183] has become the most popular approach for building vascularized constructs.

In skin tissue engineering, bioprinting dermis equivalent layers containing both HUVECs and dermal fibroblasts is a commonly used process for creating vascularized skin analogs [52, 57, 184, 185]. Fibroblasts not only modify the surrounding environment by ECM deposition but also secrete factors to stabilize *de novo* microvessels, and a variety of angiogenic factors such as VEGF, TGF-β1, interleukin-8 (IL-8) and bFGF [186] to support EC survival, growth, migration and endothelial network formation [187]. In the presence of dermal fibroblasts, ECs have been shown to form denser and more homogeneous tubular networks [17].

### Printability assessment

After selecting a bioprinting technique and the ink components, it is crucial to optimize the ink formulation to ensure a successful bioprinting process. This requires assessing printability, which refers to the ability of a material to be effectively printed under different printing conditions, resulting in the desired resolution and fidelity. Printability will directly influence the appearance, morphology, and mechanical properties of a skin tissue analog [188, 189].

Extrusion-based bioprinting relies heavily on the rheological properties of the bioink to initially assess the overall printability. An ideal ink for extrusion-based bioprinting exhibits shear-thinning behavior, which reduces viscosity under high shear stresses to facilitate smooth extrusion and lower cellular stress [190]. Cells can be modeled in the fluid as particles that occupy a volume fraction of the material, thus affecting the rheology and printability [91, 191, 192]. Key rheological parameters include yield stress, storage modulus (G′), loss modulus (G″), and damping factor (tan δ). The yield stress indicates the minimum stress needed for a bioink to flow and be extruded through a printhead [193]. G′ and G″ are common parameters that indicate a hydrogel’s stiffness and overall viscoelastic properties [194, 195]. Tan δ values between 0.25–0.45 [196] are normally preferred for good shape fidelity [197]. Recovery and thixotropy tests can also be performed to simulate the pre-, during, and post-extrusion conditions [198, 199]. Rheological tests enable fluid dynamics modeling to predict the shear stresses in the printhead or fluid velocity [200]. Sterilization methods may also impact the rheological properties and affect printability [201]. Printability assessments involve evaluating the minimum extrusion pressure, filament formation, planar organization, and layer stacking capabilities [202]. Quantitative measures, such as the printability index [202], uniformity ratio [196], and spreading ratio [203], are used to assess shape quality. The effect of gravity can be evaluated through the filament collapse test [204], and the achievable resolution can be quantified *via* the filament fusion test [199]. The integrity factor (I), indicating the ratio of printed sample height to CAD design height, aids in evaluating the ink's ability to form multi-layer structures [205]. Researchers have been working to establish correlations between rheological properties and printability outcomes to define printability optimal printing windows [204, 206]. Post-printing evaluation should include cell viability, proliferation, differentiation, and matrix remodeling potential. Printability requirements for microfluidic bioprinting are similar to those for typical extrusion-based bioprinting. To ensure precise control over droplets, bioink flow and structure formation, the ink material must have an appropriate viscosity to be handled by the microfluid channels in the printhead and should also exhibit shear-thinning properties. The size of the microfluid nozzles or channels should be compatible with the ink material to be extruded without clogging.

For DLP [207] and VBP [208], printability heavily relies on the photochemical properties of the bioinks. The ideal ink with good printability should exhibit rapid and controlled photo cross-linking with significant curing depth and high efficiency [209]. This can be optimized by balancing polymer concentration, light exposure, and free-radical production to maintain high cell viability while achieving the desired cross-linking.

In DLP, high printability is achieved with low-viscosity inks that allow easy removal of uncross-linked solutions between layers [134]. However, very low viscosity can cause cell sedimentation, affecting print homogeneity. This can be mitigated by adjusting bioink viscosity or adding platform agitating [210]. Print resolution and fidelity in DLP can be influenced by light scattering, non-confined photopolymerization, cross-linking efficiency, and the presence of cells [211, 212]. Fidelity is often evaluated using simple shapes like triangles and circles [211].

In VBP, printability depends on bioink viscosity and structural integrity. The photochemistry of the material is crucial, as reaction kinetics and mechanisms directly affect printing time, with shorter durations preferred. Optimizing polymer and photo initiator concentrations is essential for good resolution. Complex shapes, such as spirals and branched structures, are normally used to optimize VBP printing parameters [61].

### Current examples of bioprinted vascularized skin tissue analogs

Bioprinting layered multi-material constructs offers advantages for creating vascularized skin tissue analogs. While many bioprinting studies have demonstrated cell compatibility in the developed bioinks by culturing dermal cells, only limited studies have successfully bioprinted skin analogs with integrated vascular networks or demonstrated their vascularization potential.

**Figure 2:**
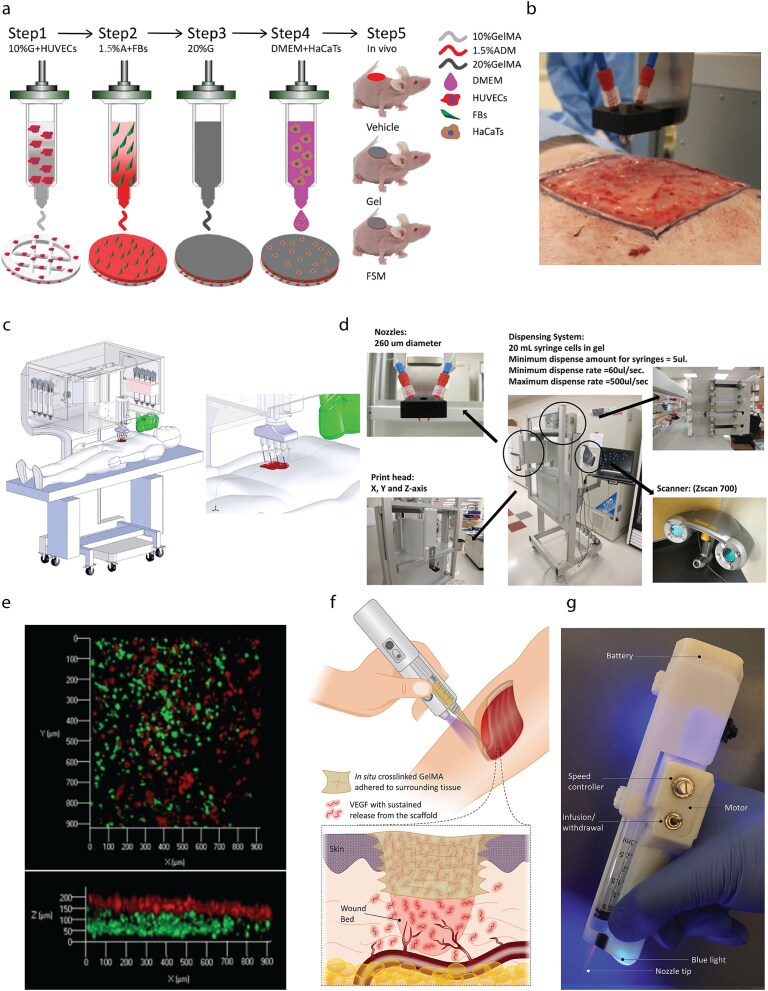
Different bioprinting techniques used for engineering skin tissue analogs with vascularization potential. (a) A schematic representation of extrusion-based bioprinting for fabricating a multi-layered skin substitute and its transplantation *in vivo*. Adapted from [213]. (b–e) Example of an *in situ* inkjet-based bioprinter for wound healing. (b) An inkjet bioprinter in the operation room. The bioink is printed using customized nozzles directly onto the wound area (porcine model). (c) Schematic representation of skin bioprinter to be used in an operation room. (d) Photo of skin bioprinter system with different components including nozzles (260 μm in diameter), dispensing system, print head and scanner. (e) Bioprinted layered fibroblasts and keratinocytes. Adapted from [49] (CC BY 4.0 license). (f and g) *In situ* bioprinting of a VEGF-eluting hydrogel for wound healing applications. (f) Schematic representation of an *in situ* bioprinting method. GelMA precursor is mixed with VEGF and extruded using a handheld device; the solution is then cross-linked on the wound site using a UV light integrated into the device. VEGF released from hydrogels can promote angiogenesis in the wound bed to enhance the quality and rate of healing. (g) Handheld device used in the study. Adapted from [32] (CC BY NC ND 4.0 license). *G* gelatin, *A* alginate, *FB* fibroblast, *DMEM* Dulbecco’s modified Eagle’s medium, *FSM* functional skin model

Extrusion-based bioprinting is cost-effective, with relatively simple ink selection, and offers advantages in printing layered skin-like structures [6, 73, 100, 185, 213–215]. Due to limited printing resolution, the vascularization potential of extrusion-printed constructs is often achieved through bottom-up processes such as vasculogenesis and angiogenesis. For example, a heterogeneous full-thickness skin model was developed by first bioprinting a 10% GelMA layer containing HUVECs. A dermis-like layer containing human fibroblasts (FBs) and an acellular dermal matrix (ADM) was then printed above it. After printing, a further layer containing 20% GelMA and HaCaTs was seeded on top of the construct to mimic the epidermis. After culturing *in vitro* for 5 days, this bioprinted multi-layered structure displayed epithelial regeneration, neovascularization, and wound healing *in vivo* [213] (Figure 2a).

Both extrusion and droplet-based bioprinting are scalable and can be used for *in situ* clinical settings (Figure 2b–g). Fibrinogen-based inks have been used for *in situ* inkjet bioprinting using a porcine model, with results showing that the deposited bioprinted fibroin/collagen hydrogel facilitated faster wound closure (complete closure at week 8) compared to an untreated group (Figure 2b–e) [49]. In another *in situ* bioprinting example, incorporation of the angiogenic factor VEGF within a GelMA ink, that was deposited as a skin analog using a handheld 3D bioprinter, promoted neovascularization and wound healing in a porcine model up to 14 days (Figure 2f and g) [32].

Light-based and VBP can also sequentially cure layers to create skin analogs containing ECs for induced neovascularization [52]. Promisingly, these printing techniques can directly fabricate fine-tuned, pre-designed hollow microchannels within printed constructs through top-down fabrication [53]. Furthermore, by adjusting the laser or light power, the degree of cross-linking can be altered to create a composite material with microscale gradients for selective cell culture [51].

### Challenges and future directions in bioprinting vascularized skin tissue analogs

The successful bioprinting of vascularized skin tissue analogs for clinical use faces several interconnected challenges, including printing speed and resolution, scalability, and sterilization. Achieving complexity and resolution mimicking the natural ECM requires significant improvements in both printing speed and resolution. Research on extrusion-based and light-based bioprinting methods has focused on balancing print quality with cellular viability, often neglecting printing speed. VBP has emerged as a promising technique, enabling the formation of constructs with vascular networks within seconds. However, more versatile inks are needed, as VBP is currently limited to specific photo cross-linkable inks, primarily those conjugated with methacrylate, tyrosine, or norbornene groups.

While large-scale bioprinting of skin tissue analogs is technically feasible, it is hindered by the need for billions of autologous cells. Efficient autologous cell expansion without loss of specific phenotypes is crucial. As construct size increases, more complex and adequate vascularization is needed. Besides the use of relevant cells, there has also been growing research into the incorporation of microvascular fragments isolated from fat tissue as vascularization building blocks in tissue engineering constructs [216–218]. These fragments are intact vessel segments that can potentially accelerate the connection (anastomosis) between host and engineered tissue. Additionally, innervation, critical for sensory perception, temperature regulation, and wound healing, has not yet been fully addressed [219–221]. Future directions will focus on bioprinting skin analogs with biochemical signaling gradients and enhancing post-implant blood vessel and nerve integration.

Scaling up bioink production while maintaining consistency and quality is another hurdle. The sterilization of bioinks also presents a significant challenge, as it may impact the material properties, leading to undesired chemical degradation or initiating secondary cross-linking reactions. Choosing appropriate sterilization methods, such as filtration, gamma irradiation, ethylene oxide treatment, and autoclaving [201, 222], is critical for preserving bioinks properties and functionalities.

## Conclusions

Bioprinting offers numerous advantages over traditional tissue engineering for creating vascularized skin tissue analogs. It enables rapid fabrication with precise control over the deposition of inks, multiple cell types, and angiogenic factors to accelerate wound healing. 3D bioprinted skin constructs show promise for treating full-thickness wounds and extensive burns. The ongoing research is focused on the design and optimization of inks tailored to the specific needs of each skin layer and the evolving capabilities of bioprinting technologies. To enable functional vascular network formation in the printed skin analogs, advanced bioinks with controlled degradation rates that mimic the natural ECM will be needed. Bioinks containing ECs and other support cells can be combined with the top-down fabrication strategies in bioprinting to achieve a vascularized construct. Additionally, thought must be given to the challenges involved in the scaling up and GMP requirements in manufacturing and the handling processes that are needed to transition from the laboratory to clinical applications. Innovations in polymer chemistry and advancements in bioprinting techniques combined with a deep understanding of skin biology and wound healing will help overcome these limitations, facilitating broader clinical use of bioprinted vascularized skin tissue analogs to significantly improve patient outcomes.
